# Reproductive Planning and the Choice of Long-acting Reversible Contraceptive Primary to Health: A Cross-Sectional Study

**DOI:** 10.1055/s-0043-1772188

**Published:** 2023-09-08

**Authors:** João Paulo Turri Brufatto, Thais Machado Dias, Natália Bortoletto D'abreu, Patricia Moretti Rehder

**Affiliations:** 1 Universidade Estadual de Campinas, Campinas, SP, Brazil

**Keywords:** reproductive planning, long-acting reversible contraceptive, primary care, clinical parameters, health education, planejamento reprodutivo, contraceptivo de longa permanência, atenção primária, parâmetros clínicos, educação em saúde

## Abstract

**Objective** Evaluate the different perspectives that involve the choice of long-acting reversible contraceptives (LARCs), the issues related to this process and the consequences of deciding one method in the women's in the primary health care (PHC) center in Sousas, a district in Campinas, SP (Brazil).

**Methods** This is an analytical cross-sectional study, it was performed at the PHC in Sousas. Data were collected through the analysis of medical records and interviews with women who live in Sousas and had the insertion of the copper intrauterine device (IUD) (D) from April 2021 to April 2022 or the etonogestrel implant (I) from May to December 2022. The study was approved by the Research Ethics Committee of the Medical Science School at the State University of Campinas (UNICAMP).

**Results** Reason for choosing this LARC: medical (D: 52%; I: 100%), easy adhesion (D: 71%; I: 67%), effectiveness (D: 55%; I: 100%). Indication by health professionals (D: 65%; I: 100%). And improvement of clinical characteristics: mood (D: 77%; I: 67%), body mass index (BMI; D: 52%; I: 33%), and libido (D: 84%; I: 67%).

**Conclusion** It is suggested that women tend to decide between LARCs when guided by their doctor or PHC health professionals, and they select LARCs because of the ease of use and low failure rates. Therefore, this study highlights how LARCs can positively interfere in the aspects that pervade contraception, such as BMI, libido, and mood.

## Resumo

**Objetivo** Avaliar as diversas perspectivas que envolvem a escolha dos LARCs, as problemáticas relacionadas a esse processo e as consequências da escolha do método na vida das mulheres no centro de Atenção Primária à Saúde (APS) em Sousas, distrito de Campinas, SP.

**Métodos** Trata-se de um estudo transversal analítico, realizado no Centro de Saúde de Sousas. Os dados foram coletados através da análise de prontuários e de entrevistas das mulheres residentes em Sousas, que inseriram o dispositivo intrauterino (DIU) de cobre (D) entre abril de 2021 a abril de 2022 ou o implante de etonogestrel (I) de maio a dezembro de 2022. O estudo foi aprovado pelo Comitê de Ética em Pesquisa da Faculdade de Ciência Médicas da Universidade Estadual de Campinas (UNICAMP).

**Resultados** O motivo da escolha: por indicação médica (D: 52%; I: 100%), pela facilidade (D: 71%; I: 67%) e pela eficácia (D: 55%; I: 100%). Da indicação por profissionais de saúde (D: 65%; I: 100%). E melhora das características clínicas: humor (D: 77%; I: 67%), índice de massa corporal (IMC; D: 52%; I: 33%) e libido (D: 84%; I: 67%).

**Conclusão** Sugere-se que as mulheres tendem a escolher LARCs quando orientadas pelo seu médico ou por profissionais de saúde da APS e optam pelos LARCs pela facilidade do uso e baixa taxa de falhas. Destaca-se como os LARC's podem interferir positivamente em aspectos que perpassam a contracepção, como o IMC, libido e estado de humor.

## Introduction

Reproductive planning, also known as familiar planning, was advocated in Brazil through Law 9.263/1996 to protect the sexual and reproductive health of adult men and women, young people, and adolescents who practice sex with or without a fixed partner. 1 2 In addition to contributing to more empowerment, reproductive planning enables women to control time between pregnancies, recover after childbirth, have safer pregnancies with lower maternal and child morbidity and mortality, and generate confidence in the decision-making of men and women regarding the type of family they want to build. 3 4 5 Thus, the need grew to offer methods of efficient and safe contraceptives for all, regardless of orientation, or sexual and gender identity.

The choice of contraceptives, in this context, became the target of several researchers and institutions internationally. 6 So, parameters like efficacy, side effects, ease of use, parity, comorbidities, information, and awareness of duration were placed as great beacons of this choice. 7 8 Finally, other beneficial effects in addition to contraception, such as improvement in weight, mood, and libido, are increasingly discussed when the topic is contraception. 9 10 11 Thus, offering the ideal method for users in each territory becomes something desired by any location with high rates of unwanted and unplanned pregnancies.

In Brazil, among the various contraceptive methods available in the Brazilian public health system (Sistema Único de Saúde – SUS), the long-acting reversible contraceptives (LARCs) are understood today as first-line methods due to their high efficacy, the similarity of results between the Pearl index in perfect and typical use, as well as for dispensing routine intervention methods, and the high continuity rates. 12 13 14

Currently, the SUS network has the availability of a copper intrauterine device (IUD), already widely used in primary health care (PHC), and the etonogestrel implant (Implanon), which was implemented through ordinance 13/2021 in the Ministry of Health. The copper IUD lasts for 10 years after insertion and may present increased bleeding and cramping as side effects. 15 As for Implanon, it has a validity of approximately 3 years, with a lower failure rate. However, it has unwanted side effects like acne and spotting. 16 Because of this, it is worth noting although the rate of use of LARCs in Brazil is low, with around 0.1% for Implanon and 1.4% for copper IUDs, they are associated with an increasing satisfaction rate of 94.7% for copper IUDs and 90% for Implanon. 17 18 19 20 This can be directly linked to the continuity of the method and, perhaps, to professionals' indication for known ones, a pattern already observed by scholars. 19 21 In this way, the dissemination of information about the LARCs, the level of satisfaction of the women, and the characteristics reported above must be studied regarding management committed to the reproductive rights of its population.

Primary health care (PHC) is part of the foundation for this adequate reproductive planning. As the main gateway to SUS, these centers contribute to the pregnancy decision based on reliable information about fertility and knowledge about the body. They also ensure that women have access to media and technologies in their territories. Additionally, Health Centers (HC) offer health care based on distance and comprehensiveness, with community and family as a focus, which is associated with clinical skills that can change the paradigms involving women's health and the choice of contraception. 22 23 24 Because of this, a program called Mais Médicos Campineiro (PMMC) was created in the city of Campinas – SP, in 2020. This strategy seeks to reorient the health model of Campinas for PHC and improve the qualification of medical professionals through specialized medical training promoted by universities such as the State University of Campinas (UNICAMP). 25

In the meantime, considering the responsibility of the government and the PHC with reproductive planning, the present work aims to evaluate the reason for the choice of implant and copper IUD by women from the Sousas HC in Campinas – SP. Furthermore, we aim to evaluate the form of acquisition of information about the contraceptive method and other aspects inherent to the process such as discontinuity rates, failures, improvements in the quality of clinical-social parameters, and side effects. Finally, this research is expected to contribute to the improvement of local and municipal reproductive planning.

## Methods

This is an analytical cross-sectional study with data collection through interviews with women from the Sousas PHC coverage area and their electronic medical records into the e-SUS. The interviews were structured by two questionnaires. The first questionnaire refers to the moment before using the method, it collected socio-demographic variables, variables on the chosen method, and characteristics of clinics. The second questionnaire refers to 6 months after the beginning of the use of the method, it collected clinical questions related to the chosen method, such as changes in the pattern of bleeding and/or dysmenorrhea, the onset of symptoms such as headache, acne, change in weight and libido. It was also asked about maintenance, failure, or expulsion of the device, if there were complications from its use, and if the woman would recommend the method to others. Both questionnaires were applied at the same time in the study. Cross-sectional refers to different moments of use of the method, previously and after 6 months of the insertion.

The women's data were separated according to the method of choice and subsequently compared with each other. We also compared the pre- and postinsertion data (IUD or Implanon). The selection of subjects consists of women registered at the PHC-Sousas who implanted a copper IUD between April 2021 and April 2022, or who adhered to the subdermal etonogestrel implant (Implanon) from May to December 2022, who agreed to participate in the research and signed the informed consent form. Implant data were collected only from May 2022, because that was when this technology was incorporated into HC respecting the criteria of the Ministry of Health of 2022. Through the database of the program Strategic Management of Materials and Medicines (GEMM), it was verified the withdrawal of 105 copper IUDs from the pharmacy at the Sousas unit, from April 2021 to April 2022.

By searching for intrauterine contraceptive device insertion (CID-10 Z30) in the electronic medical record in e-SUS, it was possible to locate 80 patients. Of these 80 women, 31 were contacted and agreed to participate in this research. It was possible to collect information from the medical records of all 31 women who had the IUD inserted in the selected months. The Implanon were inserted from May 5 to September 2022. It was possible to contact 3 of the 5 women selected, who agreed to participate in the search.

The following data were assessed using the marital question questionnaires (categorical variable): married, divorced, cohabiting, single, and widowed; age (continuous variable); comorbidities (categorical variable); body mass index (BMI, ordinal variable): reported weight (in kilograms) and height (in meters); level of education (discrete variable): in years of study; parity (discrete variable): defined by the number of previous pregnancies. We defined dependent variables as: off-cycle bleeding also known as spotting (present or absent), menstrual flow (increased, decreased, or remained the same), cramps (increased, remained constant, or decreased), discontinuation of treatment by choice, flaws in the method being used (pregnancy using the method correctly), ectopic pregnancy while using the method (presence of pregnancy outside the uterus), the onset of inflammatory pelvic disease (IPD), method satisfaction (satisfied if indicating the method to someone I know), libido (adequate, low and high), mood (happy, sad, apathetic and anxious), acquisition of information about the chosen method: health professionals, relatives or neighbors or friends, television, newspaper, and the internet. The device was considered an independent variable: IUD and Implanon.

The collected coded data were stored anonymously in a database with the Excel (Microsoft Corp., Redmond, WA, USA) software for Windows, created for this purpose. The data were allocated in tables and graphs for descriptive statistical analysis (mean, standard deviation [SD]; absolute, and relative frequency distribution). For analysis, the questionnaires were reviewed to check the readability and quality of the information, after data were organized, archived, typed, and coded. To describe the profile of the sample according to the variables under study, frequency tables were created for the variables categorical with values of absolute frequency (n) and percentage (%), and statistics descriptions of numerical variables with mean values, SD, minimum and maximum values, median, and quartiles. For the BMI, the Student-t test was used to assess statistical significance. Considering *p* < 0.05 and normal distribution or Gaussian for the BMI. For statistical analysis, the following programs were used computational systems: The Statistical Analysis System (SAS; SAS Institute INC., Cary, NC, USA) for Windows, version 9.4, as well as the Prism 5 software.

This study complied with all the principles of the Declaration of Helsinki, and Resolution 466/12 of the National Health Council, according to the guidelines and regulatory norms for research involving human beings. This study was submitted for approval by the Research Ethics Committee (CEP) of the Faculty of Medical Sciences (FCM) at UNICAMP and by the Research Commission of the DTG/CAISM under the number 59440022.5.0000.5404.

## Results

### Descriptive Variables Referring to the Cross-sectional Study

Tables 1 and 2 below show the frequency and descriptive statistics of the categorical variables on the socioclinical, data to characterize the total copper IUD sample ( *n* = 31) from 105 patients identified and the Implanon sample ( *n* = 3) from 5 patients identified in the GEMM. This discrepancy in numbers may be due to some reasons: It is possible that more than one IUD was used by each user because there is a percentage of women who remove the device and end up reinserting it afterwards, or it is possible that an IUD was discarded due to contamination during the procedure. Additionally, it is hypothesized that when recording the ICD-10 of the procedure in the medical record, the professional made a mistake in the registration, inserting another CID different from the Z30. Of all interviewed women, only one was without the method at the time of the questionnaire.

**Table 1:** Descriptive analysis of categorical variables

Copper IUD					
Humor	Frequency	Percentage	Libido	Frequency	Percentage
Happy	24	77	Adequate	26	84
Sad/anxious	7	23	Inadequate	5	16
**Menstrual flow**	**Frequency**	**Percentage**	**Cramps**	**Frequency**	**Percentage**
Increase	19	61	Increase	16	52
Decrease	3	29	Decrease	2	6
Constant	2	10	Constant	13	42
**Indicate**	**Frequency**	**Percentage**	**Marital status**	**Frequency**	**Percentage**
Yes	30	97	Married/living together	20	65
No	1	3	Single/divorced	11	35
**Implanon**					
Happy	2	67	Adequate	2	67
Sad/anxious	1	33	Inadequate	1	33
**Menstrual flow**	**Frequency**	**Percentage**	**Cramps**	**Frequency**	**Percentage**
Increase*	1	33	Increase	0	0
Decrease	0	0	Decrease	1	67
Constant*	2	67	Constant	2	33
**Indicate**	**Frequency**	**Percentage**	**Marital status**	**Frequency**	**Percentage**
Yes	3	100	Married/living together	2	67
No	0	0	Single/divorced	1	33

**Abbreviation:** IUD, intrauterine device; Implanon, etonogestrel implant. **Notes:** *Spoting percentage 50%, amenorrhea 0%, and menstrual irregularity 50%; n: numbers of women.

**Table 2:** Descriptive analysis of categorical variables relevant to choice and indication

Copper IUD					
Reason for choice					
**Effectiveness**	**Frequency**	**Percentage**	**Easy access**	**Frequency**	**Percentage**
No	14	45	No	9	29
Yes	17	55	Yes	22	71
**No hormones**	**Frequency**	**Percentage**	**IFNN**	**Frequency**	**Percentage**
No	14	45	No	26	84
Yes	17	55	Yes	5	16
**Doctor**	**Frequency**	**Percentage**	**Collateral effects**	**Frequency**	**Percentage**
No	15	48	No	25	81
Yes	16	52	Yes	6	19
**Acquisition of information about the method**					
**HP**	**Frequency**	**Percentage**	**FN**	**Frequency**	**Percentage**
No	11	35	No	16	52
Yes	20	65	Yes	15	48
**Family**	**Frequency**	**Percentage**	**TIJ**	**Frequency**	**Percentage**
No	28	90	No	25	81
Yes	3	10	Yes	6	19
**Implanon**					
**Reason for choice**					
**Effectiveness**	**Frequency**	**Percentage**	**Easy access**	**Frequency**	**Percentage**
No	0	0	No	1	33
Yes	3	100	Yes	2	67
**No hormones**	**Frequency**	**Percentage**	**IFNN**	**Frequency**	**Percentage**
No	3	100	No	3	67
Yes	0	0	Yes	1	33
**Doctor**	**Frequency**	**Percentage**	**Collateral effects**	**Frequency**	**Percentage**
No	0	0	No	2	67
Yes	3	100	Yes	1	33
**Acquisition of information about the method**					
**HP**	**Frequency**	**Percentage**	**FN**	**Frequency**	**Percentage**
No	0	0	No	2	67
Yes	3	100	Yes	1	33
**Family**	**Frequency**	**Percentage**	**TIJ**	**Frequency**	**Percentage**
No	3	100	No	3	100
Yes	0	0	Yes	0	0

**Abbreviations:** FN, friends or neighbors; HP, health professional; IFFN, indication of friends, family, and neighbors; IUD, intrauterine device; Implanon, etonogestrel implant; TIJ, television-internet-newspaper.

### Numerical Variables Referring to the Cross-sectional Study

Table 3 demonstrates the analysis of numerical variables of parity and evolution of women's BMI to copper IUD and Implanon ( Table 4 ).

**Table 3:** Data on patients' BMI

Variable	n	Mean	SD	Min	Q1	Median	Q3	Max
Copper IUD								
Pregnancies	31	2	1,14	0	1	2	2	5
BMI before	31	28.7	5.6	18	24.7	29	32.25	46
BMI after	31	27	6.2	18	23	26	30	42
**Implanon**								
Pregnancies	3	2	2.65	0	1	1	5	5
BMI before	3	28	2.64	25	25	29	30	30
BMI after	3	27.6	3.05	25	25	27	31	31

**Abbreviation:** BMI, body mass index; IUD, intrauterine device; SD, standard deviation. **Notes:** 1: before contraception choice; 2: after contraception choice.

**Table 4:** Data from medical records

Data from medical records	n	Expulsion	Removal	Pregnancy	IPD	Perforation
Copper IUD	80	4%	5%	0%	0%	0%
Implanon	5	0%	0%	0%	0%	0%

**Abbreviations:** IUD, intrauterine device; Implanon, etonogestrel implant; IPD, inflammatory pelvic disease; n, number of women.

**Fig. 1:**
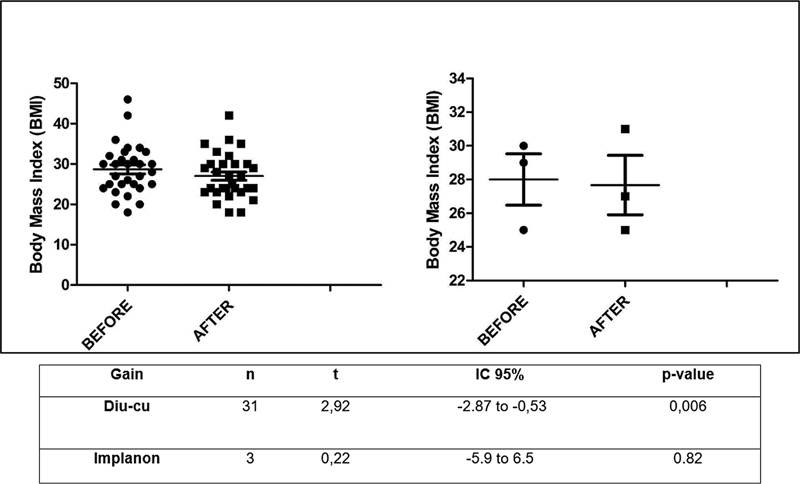
Data on body mass index (BMI) before and after the onset of copper intrauterine device (IUD) on the left and etonogestrel implant (Implanon) on the right.

The statistics are shown in Fig. 1 .

## Discussion

These results show that women choose their method according to their doctors' guidance (D: 47%; I: 77%). Additionally, the choice of these methods also involves the acquisition of information on the effectiveness of the technique through the Pearl index (D 77%; I 88%) and the ease of actual use (D: 71%; I: 67%). Furthermore, these women's rates of recommendation by others are high (D: 97%; I: 100%). Another interesting point is the fact that women's choice of LARCs involves more advice from health professionals (D: 65%; I: 100%) than the internet, newspaper, and television (D: 0%; I: 0%), in addition to the indication coming from friends or neighbors being median (D: 48%; I: 0%), and the indication coming from family members being low (D: 10%; I: 0%). Furthermore, there is an important decrease in BMI of around 2.7 for copper IUD users, with statistical significance, and of 0.22 for Implanon, with no statistical significance; improvement or maintenance of adequate self-reported libido (D: 84%; I: 66.7%) and improvement or maintenance of self-declared happy mood (D: 77%; I: 66.7%). It is also worth noting that most women in both groups were multiparous and were married or had a partner when they started using the methods, as already observed in other previous studies. 17

Data on the choice of method can be related to worldwide scientific evidence. One of these studies, the contraceptive choice project, reveals how counseling and education about contraception promoted by health professionals themselves can increase the use of LARCs and improve the reproductive planning of a given population. 8 In this sense, to the detriment of other forms of information transmission, campaigns performed by PHC professionals themselves in their territories can increase the use of LARCs by women. 26 27 28 29 Additionally, false contraindications and technical unpreparedness of physicians for insertion are also barriers to the use of copper IUD and Implanon. 30 31 32 In the present study, it is believed that the technical competence of residents in family medicine is associated with better access to inserters, due to the bond and comprehensive care obtained in PHC and the use of competent tools by family physicians. This can justify the high correlation between the use of LARCs and medical advice and acquisition of information by health professionals at the Sousas PHC ( Tables 2 ). 22 23 32 34

The data discussed above are very relevant when we add them to the data also present in the survey on the low rate of contraceptive indication by friends, neighbors, and family members in the choice of LARCs ( Tables 2 and 4 ). As several researchers have already highlighted, consolidated public policies that encourage the use of LARCs by counseling the population about their action, duration, effectiveness, and adverse effects are the future of reproductive planning. 31 32 35 36 Thus, projects such as the National Policy for Population Education in Health in the SUS (PNEPS-SUS), and policies of permanent and continued education in health for professionals, combating disinformation through the HC in the users' territories may favor the onset of LARCs. 37 38 39 40 41 42

There are other data discussed in the present work that refer to the IUD and the technical capacity of the PMMC's Family and Community Medicine residents. Expulsion rates of 4%, post-insertion PID of 0%, perforations of 0%, and pregnancy rate of 0% were obtained in the analyzed time interval. Furthermore, among the women in the study, 51% reported an increase in cramps and 61% an increase in bleeding after the insertion of the copper IUD. Such data are in line with the literature denoting the expertise of family doctors. 3 18 43 The literature also demonstrates that approximately 5 to 15% of women do not adapt to the bleeding pattern of copper IUD within 6 months of follow-up. 44 The present study, however, observed that only 5% of the women in the study requested the removal of the copper IUD due to the intense flow of bleeding, and 75% of them would still recommend it to acquaintances. Thus, it is postulated that if the Mirena—a hormonal IUD that can reduce the flow of uterine bleeding—was offered in the PHCs, there would be a good acceptance of the women who did not adapt to the copper device. 45 This study also postulated, with a 0% of expulsion, perforation, IPD, and pregnancy, that PMMC residents would probably also correctly indicate and insert the hormonal IUD, as they did in the case of the copper IUD.

A point of great importance that the present work sought to discuss is the influence of LARCs in changing other aspects of health besides reproduction. As we can see in Table 1 , both copper IUD and Implanon had beneficial effects on the mood state self-referred to as “happy”, and libido self-referred to as “adequate,” which is similar to other studies. 10 11 It is hypothesized that this happens due to the security that the method offers in avoiding pregnancies, making these women have greater control over planning their lives and avoiding situations of physical, mental, and financial violence. 43

Another great data acquired in Table 3 was the improvement in the BMI of the women who opted for the copper IUD and not of those who opted for Implanon at 6 months of use, which is consistent with the literature. 46 47 This is probably due to the large number of women who previously used the quarterly injectable as a contraceptive, a method known to cause weight gain, as well as the fact that LARCs are associated with changes in lifestyle and consequent improvement in eating behavior over time. 47 48 However, further research is still needed to better quantify these data.

In this context, other data of notorious interest are the paradigms that involve the level of satisfaction with LARCs, since this is associated with the maintenance of the methods by many authors. 12 14 19 49 50 This relevance is part of the possibility of using the best specific method for each population by understanding the factors that would lead women to discontinue the method, for example. The present research suggests that satisfaction, analyzed by questioning the recommendation of the method to others, is not always linked to the continuity of the treatment despite being often related. Thus, we had withdrawal rate of approximately 5%, a pattern similar to that of the international literature, with a counseling rate of 97% for the copper IUD and 100% for Implanon. 51 52 53 Given this data, the satisfaction with these methods, in addition to involving several issues such as effectiveness, the expectation regarding the product, and the service quality attributes, may also not always correlate with continuation of treatment. 19 54 55

Therefore, the present work postulates that adequate global assistance and satisfaction rates provided by the family physician during women's health consultations were higher than in the literature. 30 It is worth highlighting the need for further research to define whether satisfaction with the method is directly related to its continuity.

The Sousas district has a large population that does not depend exclusively on the SUS. As a result, we obtained a small number of women in both samples, and a control group that did not opt for LARCs was not included, which can create confounding biases. We had some limitations in data acquisition: ICD insertion errors, changes of address and phone number, high social vulnerability with low access to healthcare, and the small number of professionals performing Implanon insertion.

In 2020 and 2021, the whole world faced the COVID-19 pandemic, which had a multidimensional impact on health and, therefore, affected some data related to reproductive planning. This partly explains the worsening of the indicators studied from 2020 onwards.

## Conclusion

The data presented suggest that women choose methods according to the guidance of their physician or health professionals and opt for LARCs due to their ease of use and low failure rate. Additionally, the possible improvement of important clinical parameters for the general wellbeing of women, such as libido, mood, and BMI related to LARCs, is highlighted. Furthermore, family medicine residents make a correct indication and insertion of LARCs, with adequate advice and assistance provided. Further research and a more longitudinal outlook are needed to detail other aspects relevant to the LARCs in the Sousas territory, as well as to be able to externalize our results to other realities.
